# Maintaining Distance and Staying Immersed: Practical Ethics in an Underresourced New Born Unit

**DOI:** 10.1177/1556264619835709

**Published:** 2019-05-16

**Authors:** Joyline Jepkosgei, Jacinta Nzinga, Jacob McKnight

**Affiliations:** 1 KEMRI-Wellcome Trust Research Programme, Nairobi, Kenya; 2 Nuffield Department of Medicine, University of Oxford, UK

**Keywords:** situated ethics, neonatal, LMIC, practical ethics, positionality

## Case Study

### Country Context (Including Health Features)

Within sub-Saharan Africa, Kenya is ranked relatively highly for access to health care services, but its public health system is under considerable stress due to increased demand, high patient to staff ratios, and the complete devolution of health services to largely autonomous County health teams (Wakaba et al., 2014; World Health Organization [WHO], 2006). New Born Units (NBUs) dealing with sick infants are particularly under strain, with inadequate availability of basic drugs and equipment and a ratio of 15:1 patients to nurses, while in high-income countries such as the United Kingdom, the recommended ratios are 4:1 (Aluvaala et al., 2015; British Association of Perinatal Medicine, 2001; Murphy et al., 2018). The lack of effective care can be directly linked to significant neonatal mortality in Kenya (Gathara et al., 2011). In 2014, the neonatal mortality was at 22 deaths per 1,000 live births (Kenya National Bureau of Statistics, Ministry of Health/Kenya, National AIDS Control Council/Kenya, Kenya Medical Research Institute, National Council for Population and Development/Kenya, and ICF International, 2015), which indicates a need for significant progress before Kenya achieves the *Every Newborn* action plan target of 10 or less neonatal mortality per 1,000 live births by 2035 (WHO & UNICEF, 2014).

### Description of the Study/Research/Situation in Which the Ethical Issues Arose

The Health Services that Deliver for Newborns (HSD-N) project is supported by a multidisciplinary team with experience in ethnographic work, epidemiology, and health systems. The project aims to investigate the projected need for neonatal inpatient services; what existing infrastructure and human resource capacity is available supporting access for this population; utilization of these services; and the quality of existing nursing care services. The latter aim is supported by an ethnography of neonatal nursing. This research was undertaken at three of Nairobi’s NBUs over a period of 18 months, beginning early in 2015. Two of the three researchers were based in Kenya, while one was primarily based in the United Kingdom. All three researchers are social scientists, they were involved in the data collection, and all research was coordinated through the KEMRI-Wellcome offices in Nairobi.

### Case Vignette

The ethical implications of this setting are significant: The infants in the NBUs were extremely vulnerable as were their mothers who were frequently overwhelmed by feelings of despair and anxiety. Furthermore, this work took place during a period of nationwide health worker strikes which strained relationships between health workers and their employers. Consequently, nurses were unhappy and stressed and further were potentially at risk of losing their jobs if they were seen to complain too much. Under these conditions, our aim with regard to ethics was to be as prepared as possible while also treading carefully and remaining reactive. Unfortunately, we still encountered two major ethical dilemmas: the first concerned our respondents’ apparent willingness to delay work to speak to us, thus creating a fundamental ethical issue in putting infants at increased risk (World Medical Association, 2013); and the second, the potential harm resulting from being asked to help in medical tasks having quickly been accepted as “one of the team” (Department of Health, Education, and Welfare; National Commission for the Protection of Human Subjects of Biomedical and Behavioral Research, 2014). Both issues were made more difficult to manage by the emotional toll posed by ethnographic immersion into such a resource-limited, high-stress environment.

The application passed through three ethics review committees prior to the start with significant feedback and editing of the protocol. One of the two ethics committees that reviewed the study was formed of senior researchers with great experience in research ethics. Upon receiving approval, the first phase of interviewing began. We determined that it would be best to speak to senior nursing trainers, and leaders of nursing organizations first, because in addition to providing us with a managerial and policy-level view, they also helped us secure access.

Following the interviews with the senior managers and leaders, we managed to gain access to the hospitals. After gaining verbal permission from each hospital manager or CEO, we began interviewing senior members of the nursing staff. Interviews with nursing “in-charges” (matrons) were conducted in the nursing station or a private office, and this did not seem to interrupt the flow of work, because they were not involved in direct clinical work. We were then allowed to speak to more junior members of staff and began interviewing frontline nurses.

One of the first topics to be raised in these interviews was the ratios of nurses to babies—very much lower than international or even national standards. Our ethnographic interviewing methods generally demanded that we spoke to our interviewees for 40 to 60 min, and we soon realized that we were taking active nurses away from the wards to interview them. The nurses seemed willing to do this, but we felt uncomfortable and so asked if we could interview them after the shift. It seemed clear that this would not be possible given nurses’ other responsibilities, and so we tried to ensure that our interviews were conducted during well-staffed morning shifts, or during very quiet moments. We had not anticipated such challenges in finding appropriate slots for interview. It would have been easier to agree with the nurses and accede to interviewing them during the shift, but this felt wrong. It was difficult to explain our concern to the nurses while avoiding the suggestion that they were being negligent in their willingness to be interviewed during the shift. Our concerns were based on one of the most basic ethical principles—to protect those involved in the research from harm (Department of Health, Education, and Welfare; National Commission for the Protection of Human Subjects of Biomedical and Behavioral Research, 2014)—but our challenge was to also manage the relationship with the subjects of our research while doing this. Such difficulties have been encountered by researchers elsewhere and it is important that we stay alert to the challenge of situated ethics issues such as these (Molyneux & Geissler, 2008). In our case, we shared our experience in the larger research team and our experience led to the adoption of a policy that offers financial compensation for out-of-hours interviews.

After gaining a level of trust from the nurses, and understanding of their work, we began observation of the shift work. This was critically important, as it provided a great deal of context and ethnographic detail to what we had learned from interviews. Unfortunately, it also put a great deal of strain on one team member, who found it difficult to navigate this particularly stressful environment. First, the strains and emotions of the NBUs we studied wore heavily on the observer, and witnessing the death of infants and failed resuscitations was distressing. Of course, this was helpful for the research, as we began to witness if not directly feel, the “emotional labour” of nurses. However, we were ill prepared for the stress of the NBU and had not considered how hard the research would be.

A further problem was encountered in being asked by nurses for help. At first, this was easy—It was not ethically problematic to be asked to fetch certain members of staff or folders. However, it became harder to manage when we were asked to help with medical care. During one emergency, a nurse asked one of us to turn the dial on the oxygen machine. The researcher felt this was beyond their ethical mandate and was able to find help on this occasion, but in other pressing situations, we felt that providing some assistance was ethically reasonable. For example, one of our researchers helped cup-feed the abandoned babies who did not have a mother to do it for them. In hindsight, we should have perhaps predicted where our immersion into the ward may lead, but we did not react quickly enough to prevent a particularly problematic event. The nurses we worked with were often overwhelmed—not only by the high patient to nurse ratios in this setting but also with the difficulties of commuting and managing responsibilities at home. This was evidenced by the fact that nurses frequently arrived late to work and would “rest” (sleep) when the pressure eased. Unfortunately, on one relatively quiet night, a nurse who was meant to relieve an afternoon shift nurse, phoned to inform them that they were running late. The afternoon shift nurse cited major demands at home and announced they could not wait, and so left only our non-medically trained researcher to watch the ward. We were unprepared to deal with this event and feel very fortunate that no harm seemed to result. Striking the correct balance between ethnographic immersion into the culture of nursing and maintaining sufficient barriers to protect infants was thus very challenging. The challenges posed by the use of ethnography in similar contexts has been noted by researchers, for example, Lichtner (2014) shares her experience of fieldwork with vulnerable patients; she highlights the constant presence of situated ethics such as “calls for assistance” by patients or staff. It is therefore important that we reflect on our findings and continually improve our ethical approach to account for this.

### Ethical Issues Arising

#### Frontline interviewing as obstructive to care

We had presumed that nurses would never put patients at risk to participate in the research, but this was not a simple matter. The nurses’ understanding of “busy” was quite different to our own, and we soon realized that nurses were willing to leave the ward at times where we felt patients might be at risk. Avoiding harm, especially to vulnerable patients, is a key tenet of ethical research practice (Department of Health, Education, and Welfare; National Commission for the Protection of Human Subjects of Biomedical and Behavioral Research, 2014) and so we needed to develop our own systems for ensuring the interviews did not result in a reduction in the quality of care.

#### Medical responsibility and liability

Our medically unqualified researchers were asked to help with simple administrative tasks such as fetching people or things, and this was fairly unproblematic. But being asked to help with emergencies and direct infant care raises important issues around accountability and duty of care. As described above, on one occasion, a nurse who had clearly come to see our researcher as one of the team left the shift entirely, leaving only our researcher on the ward with 12 sick infants. Thus, while we were driven to reduce barriers as part of our ethnographic approach, this presented challenges to our ethical practice in this environment (Hoeyer, Dahlager, & Lynoe, 2005).

### Conclusion

The questions raised above were not addressed in the ethical review and we failed to identify them as concerns quickly enough. However, in response to the ethical challenges we encountered, we did conduct debrief meetings within the research team where we shared our experiences and agreed on possible actions. Through this mechanism, we were able to address some of the issues. Two of the researchers also attended an emotional management course aimed at helping researchers deal with emotional distress.

Throughout the study, we provided feedback to each of the study hospitals, where we also discussed some of the ethical challenges that arose during data collection. We hope to learn from our experience and are working with our ethics team to ensure future reviews cover these topics. We also recognize the need for reflexivity. However, while a generic sense of “keeping distance” was a consideration, how it applied to the specifics of the research only became apparent through the “doing” of the research.

In addition, our experiences highlight the fact that the ethics of conducting health policy and systems research are relatively underexplored in low-and-middle income countries (Molyneux et al., 2016). While there is an emerging understanding of the need for a more “situated ethics,” guidance specific to context and particular environments is lacking, and it seems important to both to share and learn from our experiences and also to create systems that allow researchers to react more quickly.

## Suggestions for Commentary Topics 1 and 2

Fairness toward co-researchers or participants

Research integrity and responsible conduct of research
